# Hybrid Temperature and Stress Monitoring of Woven Fabric Thermoplastic Composite Using Fiber Bragg Grating Based Sensing Technique

**DOI:** 10.3390/s20113081

**Published:** 2020-05-29

**Authors:** Changhao Chen, Qi Wu, Ke Xiong, Hongzhou Zhai, Nobuhiro Yoshikawa, Rong Wang

**Affiliations:** 1 State Key Laboratory of Mechanics and Control of Mechanical Structures, Nanjing University of Aeronautics and Astronautics, Nanjing 210016, China; chenchanghao@nuaa.edu.cn (C.C.); zhaihongzhou@nuaa.edu.cn (H.Z.); wrong16@nuaa.edu.cn (R.W.); 2 Institute of Industrial Science, The University of Tokyo, 4-6-1 Komaba, Meguro-ku, Tokyo 153-8505, Japan; nobyoshi@iis.u-tokyo.ac.jp

**Keywords:** temperature monitoring, stress monitoring, thermoplastic composite, woven fabric composite, fiber Bragg gratings

## Abstract

Process monitoring of woven fabric thermoplastic composite is crucial to enhance the quality of composite products. In this work, a new fiber Bragg grating based technique was proposed to achieve hybrid temperature and stress monitoring according to the changes of wavelength and reflectivity, respectively. The sensor head consisting of a pre-annealed fiber Bragg grating and a steel capillary was properly designed to overcome the challenge of high forming temperatures up to 332 °C, complex woven structure, and high forming pressure of 2 MPa, which hinder the use of the conventional fiber Bragg grating sensor during the forming process. The forming temperature changes of thermoplastic composite in the heating, dwelling, and cooling phases can be precisely measured by the proposed sensor head after using a curve-reconstruction algorithm based on cubic polynomial fitting. The measured difference from the reference thermocouple is 2.92 °C, averaged from three sets of repeated experiments. Meanwhile, the change of the residual stresses of the composite can be illustrated by using the micro-bending-caused optical power loss of the fiber pigtail commencing at the glass-transition temperature in the cooling phase. The decrease of grating reflectivity that was equivalent to the optical loss was discussed by comparing to strain change detected by strain gauges and a calculated theoretical curve. These results are beneficial for developing an advanced in situ monitoring technique and understanding the forming process of the woven fabric thermoplastic composite.

## 1. Introduction

Fiber-reinforced polymers have been used widely in aerospace, transportation, and other industrial sectors owing to their advantageous performance, such as high specific strength, design flexibility, and excellent resistance against fatigue and corrosion, which are superior to those of conventional metal materials. In particular, fiber-reinforced thermoplastic polymers (FRTPs) have a vast potential market and are receiving more attention because of their shorter production cycles, higher productivity, and lower production costs.

Compression molding is a typical forming process used to manufacture FRTPs in which temperature is a crucial parameter [1,2]. The thermally induced residual stresses cause structural deformations after demolding, leading to material failure and even catastrophic failure of the whole structure [3]. Thus, in situ real-time temperature and strain/stress monitoring during compression molding are eagerly desired for investigation of the forming process and optimizing the quality of final products. Traditionally, thermocouples (TCs) and strain gauges are used to measure the temperature and strain, respectively. However, the normal type of sensors is not suitable for embedded detection as severe initial damage of the composite may be induced by their relatively large sizes. Other additional issues, e.g., the electromagnetic interference, also hinder their practical application though there are advanced TCs with small sizes [4]. Thus, both academia and industry are keen to develop a new measuring technique that can effectively monitor the composite-forming process without affecting its mechanical properties after the forming process.

In recent decades, fiber Bragg grating (FBG) sensors have been embedded into the composite to monitor the forming process, because of the advantages of small size, high-temperature resistance, and electromagnetic interference immunity. When an FBG serves as a temperature sensor embedded in composite, an encapsulating technique is usually necessary to eliminate its strain influence because of the cross-sensitivity of the FBG to the strain and temperature. Although FBG has been proven to measure temperature above 1000 °C [5,6], its application of in situ temperature monitoring in the harsh environment of the composite forming process, that has high local pressure with uneven distribution, has not been thoroughly investigated yet. Takeda et al. [7] monitored the temperature of a carbon/epoxy panel manufactured by vacuum-assisted resin transfer molding in its curing process under 120 °C with a measurement accuracy of 0.1 °C. Guo [8] measured the temperature up to 180 °C during the forming of a laminate that was made of T300/HD03 (carbon/epoxy) prepregs and had an asymmetric cross-ply stacking sequence. There are more studies on temperature monitoring of thermoset composite using encapsulated fiber Bragg gratings (EFBGs) at temperatures lower than 200 °C [9,10,11]. Mulle et al. [12] monitored a glass fiber/thermoplastic matrix laminate by embedding EFBGs at different locations throughout the thickness of the laminate, and the temperature during fast or slow cooling rates was recorded during compression molding. The maximum forming temperature measured in his experiment was 220 °C. The Bragg wavelength shift was treated as linearly proportional to the temperature in this range, although the nonlinearity between the Bragg wavelength shift and the temperature was recognized by the authors. As these existing techniques restrict the measuring temperature lower than 220 °C and measuring object to unidirectional or cross-ply composite, they cannot satisfy some specific applications under harsh conditions. For example, polyetherimide (PEI)-matrix woven fabric thermoplastic composite raises high demands on both optical and mechanical robustness of the FBG sensor head. A high temperature of 332 °C is needed to achieve the sufficient melt index of the thermoplastic matrix and ensure the molding capability of the laminate. At such a high temperature, conventional FBG exhibits an irreversible wavelength shift of approximately −0.2 nm [13] and shows a nonlinear relationship between the temperature and Bragg wavelength shift [14], leading to measurement errors. Thus, the repeatable relationship of the Bragg wavelength shift and temperature, namely optical robustness, should be ensured. The mechanical robustness of the sensor head implies a stable sealant and strong capillary. The PEI at the rubbery stage is fluid, and thus, demands a stable sealability of sealant at high temperature to prevent resin from immersing into the capillary [15]. The chosen prepregs are woven from perpendicular warp and weft yarns, and the applied pressure in the compression molding of FRTP is high, e.g., 2 MPa for PEI-matrix composite. As a result, the sensor head should be capable of withstanding high local pressure and prevent the inside FBG from distortion. All of the high processing temperatures, microwoven structure, and high pressure challenge the feasibility of the embedded sensor and obstruct the development of composite-forming process monitoring. 

Besides temperature monitoring, the strain monitoring of composite materials using FBG also has been intensively researched. Michael et al. [16] used bare FBGs to monitor strain in glass-fiber-reinforced thermoset composite laminates. Takuhei et al. [17] monitored the residual strain in thick thermoplastic composite laminates using embedded FBG sensors. More research on in-plane strain monitoring can be found in references [18,19,20]. Minakuchi [21] monitored the out-of-plane strain during the curing process of unidirectional thermoset composite laminates by embedding an FBG in the through-thickness direction as the out-of-plane shrinkage is also a key deformation [22,23,24]. However, it is very difficult to embed FBGs in woven fabric composites in the same way. In these existing techniques, strain measurement usually requires an additional sensor and/or instrument to decouple the temperature- and strain-induced Bragg wavelength shift, resulting in a redundant system and high cost. In addition, residual stresses that are usually calculated by multiplying the strain and the material modulus are not directly available owing to the sensing principle of the FBG. However, because residual stresses play a crucial role on influencing the structure deformation after demolding and onset of damage in service of the composite [3], both the academia and industry eagerly prefer a single sensor that can reflect the change of residual stresses of the composite while does not increase the burden of equipment and investment.

In this research, a hybrid temperature and stress monitoring of a woven fabric composite laminate that has glass fiber-reinforcement and PEI matrix in its compression molding process was achieved using a single FBG based sensor. The problems of high temperature, high pressure, and the influence of microwoven structure to conventional FBG-based in situ monitoring technique were solved by properly designing a pre-annealed sensor head and a data-processing method. The experimental results demonstrated the reliability of the novel sensing technique through a comparison of the temperature and strain measured by the TCs and strain gauges. The temperature curve of the composite laminate during heating, dwelling and cooling phases was precisely described, and the out-of-plane residual stress was also revealed through the micro-bending-caused optical loss of the fiber pigtail without the use of an additional instrument, which helps to understand the different states of the woven fabric thermoplastic composite in the forming process.

## 2. Sensor

### 2.1. Structure of EFBG Sensor

FBG is manufactured by writing a periodically modulated index in a fiber core. It reflects a portion of the incident light around its Bragg wavelength *λ_B_*, When an FBG is subjected to a temperature change Δ*T* or an axial elastic strain *ε*, the shift of *λ_B_* is expressed as

(1) $$\Delta \lambda_{B}=K_{\varepsilon }\varepsilon +K_{T}\Delta T,$$

where *K_ε_* and *K_T_* are sensitivities to strain and temperature, respectively. The values are 1.21 pm/με and 13.72 pm/°C to an FBG with a Bragg wavelength of approximately 1550 nm [25].

An encapsulating technique was applied on the bare FBG to isolate the strain influence and solely measure the temperature. In addition to decoupling strain and temperature, the encapsulating technique can also prevent the spectrum of FBG from distorting as the package outside can prevent transverse loads from pressing the internal grating [26].

**Figure 1:**
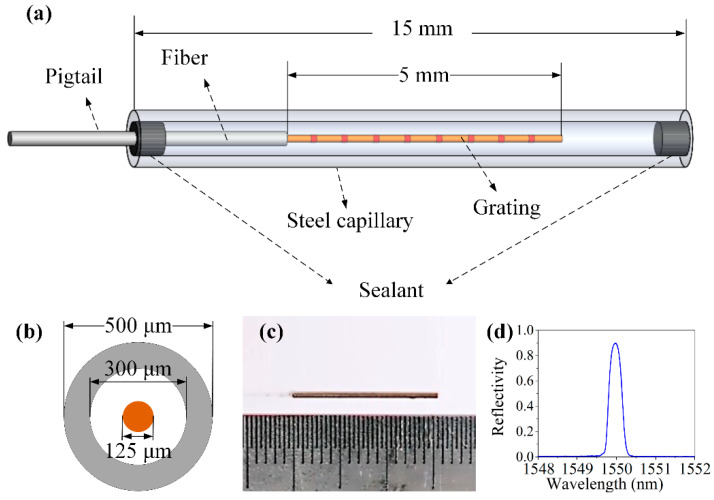
(**a**) Schematic diagram, (**b**) cross section, (**c**) photo of the encapsulated fiber Bragg grating (EFBG) sensor, and (**d**) typical reflection spectrum.

Figure 1a,b shows the schematic diagrams of the profile and cross section of the EFBG sensor, respectively, Figure 1c is a photo of the manufactured EFBG sensor, and Figure 1d is a typical reflection spectrum of an EFBG. The outside diameter of the fiber core, cladding, and coating are 9, 125, and 250 μm, respectively, and the grating length is 5 mm. A 304-steel capillary with inner and outer diameters of 300 and 500 μm, respectively, was installed out of the FBG after the acrylate coating at the grating area was removed. Removing the coating eased the alignment of the FBG and capillary under the microscope. The capillary using 304 steel with a yield strength of 205 MPa ensured the strength of the sensor head. Then, both ends of the capillary were sealed by an aluminum silicate sealant that was resistant to high temperatures up to 1300 °C. The sealant was cured at room temperature for 24 h. By using this procedure, the FBG in the capillary is hanged in the midair and consequently isolates the outside loadings, but it can effectively detect the temperature as heat capacity, and the weight of such a device is quite small.

A pre-annealing process was required to eliminate the irreversible Bragg wavelength shift of EFBG sensors at the temperature above 300 °C [27]. In this work, the pre-annealing temperature was set to 350 °C, 18 °C higher than the forming temperature of the composite. After 2 h annealing, *λ_B_* of the EFBG shifted −0.13 nm from its initial value, whereas its full width at half maxima and reflectivity were almost the same, around 0.4 nm and 0.8, respectively.

### 2.2. Calibration Results

**Figure 2:**
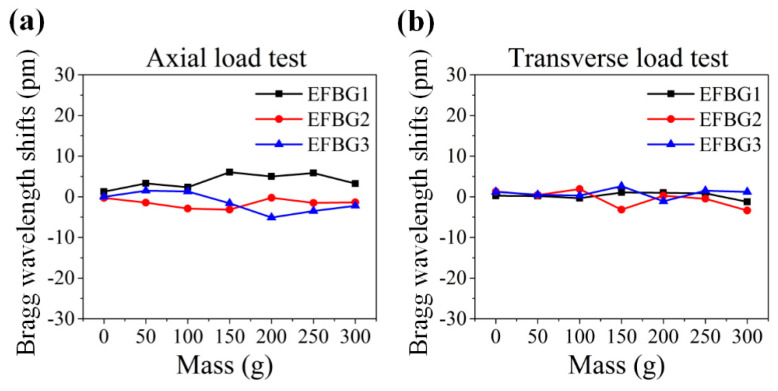
Bragg wavelength shifts of EFBG sensors under (**a**) axial load and (**b**) transverse load.

The EFBG sensor head was tested and calibrated before application. First, its mechanical performance when subjected to axial and transverse loads was tested. The Bragg wavelength shifts of three EFBG samples in these two tests are shown in Figure 2a,b. The shifts are smaller than 10 pm and are ignorable, indicating that the axial and transverse loads do not affect the EFBG sensors. In other words, strain isolation has been accomplished successfully using the encapsulating technique.

**Figure 3:**
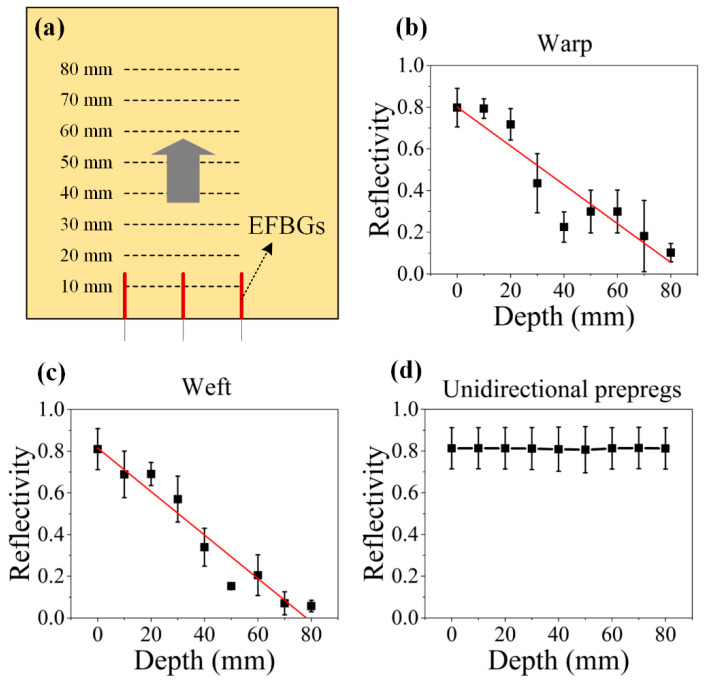
(**a**) Schematic diagram of the embedding test, and relation of reflectivity of EFBG sensors and the embedding depth (**b**) along fiber-reinforced thermoplastic polymer (FRTP) warp, (**c**) along FRTP weft, and (**d**) in unidirectional carbon/epoxy prepregs.

Second, an embedding experiment was conducted to test the performance of the EFBG by embedding the sensor into the woven fabric composite prepregs (TC1000 Design, TenCate Cetex^®^, Nijverdal, The Netherlands) and another unidirectional carbon/epoxy prepregs under 2 MPa pressure. Figure 3a is the schematic diagram of the embedding test. Figure 3b,c shows the reflectivity-versus-depth curves in warp and weft directions, respectively. In both cases, the reflectivity decreases with the increasing embedding depth. Because the metal sensor head is strong, the reflectivity decrease is predominantly due to the pigtail. When the pigtail was embedded into the prepregs, the microstructure of the woven fabric composite pressed the fiber, resulting in micro-bending. Multiple micro-bending can lead to a noticeable extra loss in optical power [28,29,30]. In Figure 3b,c, the reflectivity along weft reduced more than that along the warp because of the more warp count per meter compared to the weft count. In contrast, the reflectivity of the sensor in the unidirectional composite barely changed in Figure 3d. These results provided a reference for the embedding depth of EFBG sensors in the subsequent monitoring experiments.

**Figure 4:**
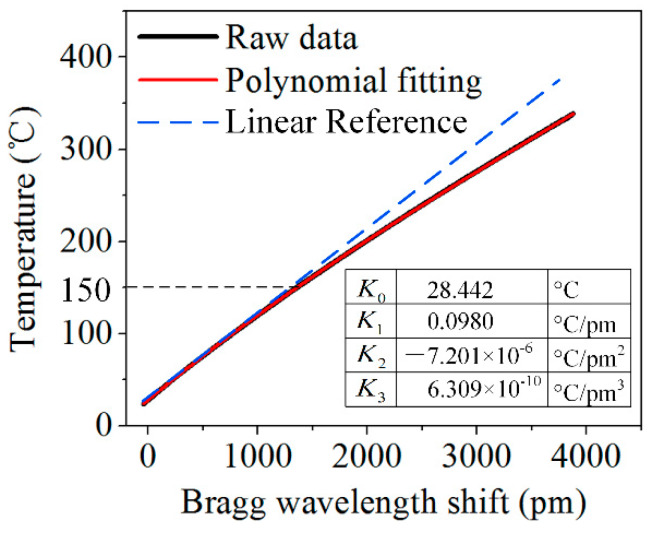
Temperature calibration using a cubic polynomial fitting equation.

Finally, temperature calibration was conducted. The EFBG sensor and TC (C060-K, CHINO, Tokyo, Japan) were heated together to 332 °C. Figure 4 presents the temperature-versus-Bragg wavelength-shift curve. It is found that the relationship between the thermally induced wavelength shifts and temperature changes is nonlinear when the temperature is approximately 150 °C. The temperature *T* acting on the EFBG sensor can be expressed by using a cubic polynomial fitting as [27]

(2) $$T=K_{0}+K_{1}\Delta \lambda_{B}+K_{2}\Delta \lambda_{B}{}^{2}+K_{3}\Delta \lambda_{B}{}^{3},$$

where *K*_0_, *K*_1_, *K*_2_, and *K*_3_ are coefficients whose values are listed in Figure 4. The average absolute difference between the fitting curve and the experimental data is 0.0017 °C, indicating the precision of the temperature measurement.

## 3. Experiment

### 3.1. FRTP Laminate

The composite laminate was manufactured from the prepregs (TC1000 Design, TenCate Cetex^®^, Nijverdal, The Netherlands) that consist of PEI as matrix and plain woven glass fibers (Glass 7628) as reinforcement. The properties of PEI and glass fiber at room temperature are shown in Table 1. The ply thickness of the Cetex^®^ prepreg is 0.16 mm. Twenty plies of prepregs with the same layup direction were used in this experiment.

### 3.2. Forming Procedure

**Figure 5:**
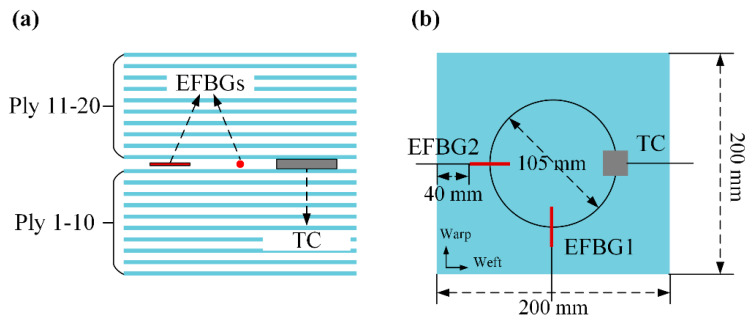
Sensors embedded in the composite: (**a**) front view and (**b**) top view.

In the temperature-monitoring experiment, two EFBG sensors and a TC were embedded in the middle of the laminate with a size of 200 × 200 mm, as shown in Figure 5. Both the EFBG sensors and TC were laid 52.5 mm away from the center. The 40-mm embedding length of the pigtails of the EFBG sensors ensures sufficient reflectivity. EFBG1 and EFBG2 were placed along the warp and weft, respectively. The head of the reference-sheet TC has a size of 7 × 7 × 0.2 (L × W × H) mm, and its lead wire has a diameter of approximately 2 mm. It is highly possible that embedding TC with large-diameter wires into the laminate will cause initial material damage, and then affects the mechanical performance of the manufactured laminate. In contrast, EFBG has better minimal invasion capability due to its diameter of 500 μm. 

In the strain-monitoring experiment, two strain gauges (KFRP-5, KYOWA, Obu, Japan) were embedded parallel to the EFBG1 to monitor the local in-plane strain in the warp directions to clarify the mechanism of reflectivity change during the entire forming process. The lead wires of the strain gauges were glued using ethyl α-cyanoacrylate, whereas the sensor head was not fixed to the target material.

The woven fabric composite with the sensors was placed in a hot press (3690, CARVER, Wabash, IN, USA). An interrogator (sm130, Micron Optics, Atlanta, GA, USA) with a sampling frequency of 1 kHz and a resolution of 0.1 pm was used to record the Bragg wavelength shift. The temperature and strain were collected using a data logger (NI9219, National Instrument, Austin, TX, USA) and static strain meter (XL2101B, XIELI, Qinhuangdao, China).

The forming protocol of the TC1000 prepregs is provided by TenCate and can be divided into three phases. In the heating phase, the target temperature was set to 332 °C. In the dwelling phase, a pressure of 2 MPa was applied to the prepregs. In the cooling phase, the laminate was naturally cooled to room temperature together with the molds.

### 3.3. Manufactured Laminate

**Figure 6:**
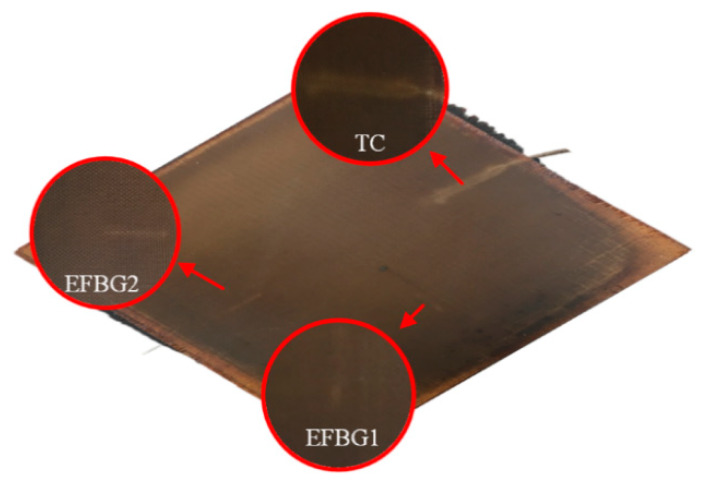
Manufactured laminate with EFBG sensors and enlarged details.

Figure 6 presents the manufactured laminate. The embedded EFBG was barely visible to the naked eye, as the surface of the composite was flat. In contrast, the embedded TC was obvious, even observed from outside. It is believed that the TC induces initial damage to the composite.

**Figure 7:**
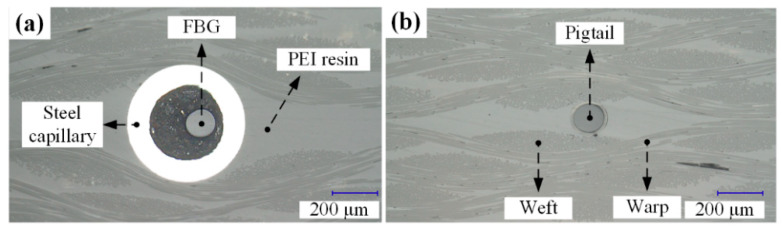
Cross section of the (**a**) head and (**b**) pigtail of the embedded EFBG sensor.

After the composite was formed, a laminate was cut, and the cross sections of the head and pigtail of the EFBG sensor were observed under a microscope. Figure 7 shows that the sensor head did not deform under the forming pressure, and the composite does not have voids, cracks, or delamination. It has been reported that resin-rich areas resulting from an embedded bare optical fiber do not affect the mechanic performance of composite significantly [31]. Therefore, it is believed that a slightly larger resin-rich area existing around the EFBG sensor head has a limited impact on composite performance. Moreover, the resin-rich area can be reduced if the size of the sensor head is optimized in the future.

## 4. Temperature Monitoring Using Wavelength Shift

### 4.1. Bragg Wavelength Shift

**Figure 8:**
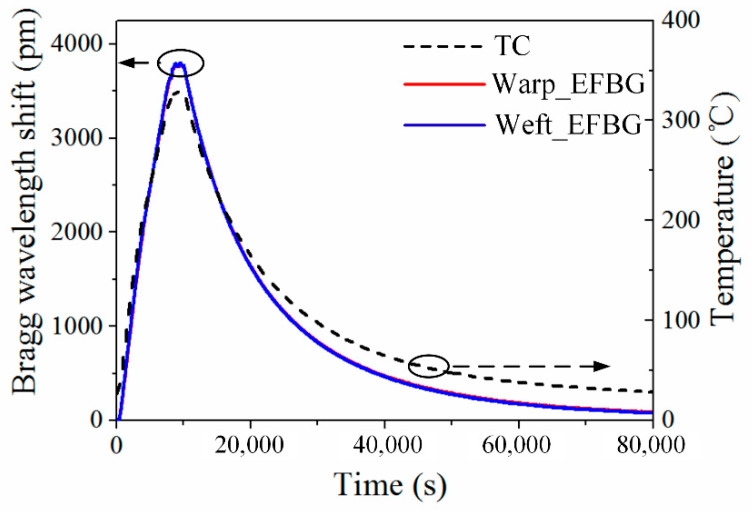
Raw data obtained from EFBGs and TC.

The Bragg wavelength shifts obtained from the EFBGs and the temperature monitored by TC in the experiment are shown in Figure 8. The data from two EFBGs is consistent with an average difference of 6 pm (i.e., the blue and red curves overlap) and has a similar trend to the temperature curve from the TC, comprising the heating, dwelling, and cooling phases of the forming process. The difference shown in Figure 8 between these curves is mainly caused by the nonlinear relationship between the temperature and Bragg wavelength shift of the EFBG. Thus, the correct temperature curves should be reconstructed by using a cubic polynomial fitting Equation (2).

### 4.2. Temperature Reconstruction

**Figure 9:**
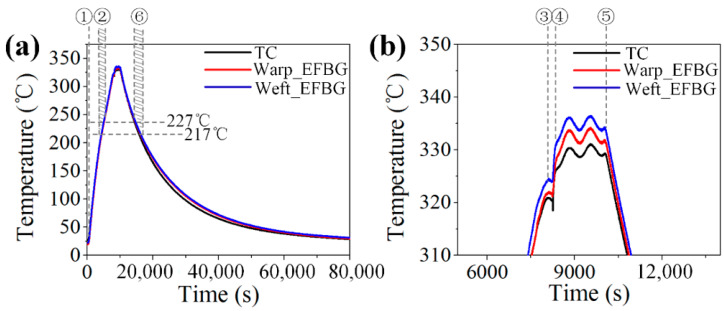
Reconstructed temperature cycles from EFBG: (**a**) entire temperature cycle and (**b**) enlarged view of the dwelling phase.

Figure 9a presents the reconstructed temperature curves from the EFBG sensors, and Figure 9b shows the enlarged view of the dwelling phase. The whole forming process can be further divided into six stages, as follows:

Stage 1: The molds of the hot press were closed to generate minimum contact between the molds and the prepregs for effective heat transfer, but no pressure was applied at this stage. The temperature rose from room temperature at a rate of approximately 2.5 °C /min.

Stage 2: After the temperature reached Tg (217 °C) in the heating phase, PEI transformed from the glassy state to the leathery state. Then, it entered a rubbery state when the temperature further increased beyond Tp (227 °C) [15]. During this period, the temperature measured by the EFBGs smoothly increased.

Stage 3: The temperature meter indicated that the hot press reached the setting temperature of 332 °C, but both the EFBG sensors and the TC showed a lower temperature of 325 °C. This was because of the insufficient contact between the molds and prepregs. The 7 °C temperature difference between the composite and the hot press indicated that it was necessary to in situ monitor the temperature during the forming process using embedded sensors. A lack of in situ monitoring technique may yield a non-negligible difference between the actual temperature and the temperature required for composite forming, and thereby affecting the final quality of the product.

Stage 4: The pressure was increased to 2 MPa to achieve full contact between the molds and the composite. Then, the temperature in the middle of the laminate rose rapidly. The temperature measured by two EFBGs and one TC was 332 ± 5 °C, which was close to the temperature of the hot press molds. This temperature difference was mainly caused by the uneven temperature distribution on the composite. Figure 9b demonstrates that the EFBG sensors could endure the high pressure in the forming process, and the small fluctuation of the temperature caused by the temperature controller of the hot press can be manifested effectively by the EFBG, showing their high time response.

Stage 5: The composite was naturally cooled while the pressure remained constant. The temperature detected by the EFBG sensors and the TC can effectively reflect the change.

Stage 6: PEI transformed from the rubbery state into the leathery state when the temperature decreased to 227 °C. It did not enter the glassy state until the temperature was lower than 217 °C. After the whole composite laminate cooled to room temperature, it was demolded.

**Figure 10:**
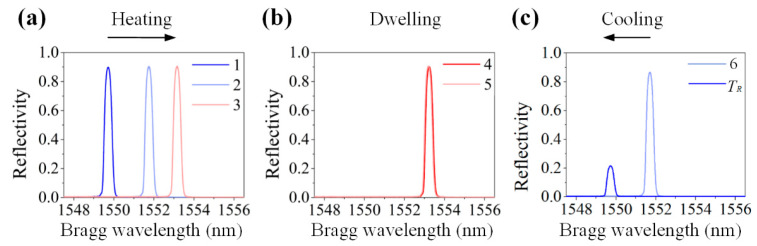
Reflected spectra in (**a**) heating, (**b**) dwelling, and (**c**) cooling phases.

Figure 10 shows the typical spectrum change in an EFBG in all six stages and at room temperature *T_R_* of 25 °C before demolding. No spectrum distortion could be observed, as the FBG spectrum in all stages shows Gaussian profiles. This demonstrates that the pre-annealing and capillary are useful to decoupling temperature and strain and preventing spectrum distortion. Although it is also apparent that the reflectivity of the sensor decreased in the cooling phase, which will be discussed in detail in Section 5, the EFBG temperature measurement is reliable, as the temperature information is solely related to the Bragg wavelength shift.

### 4.3. Repeatability Validation

**Figure 11:**
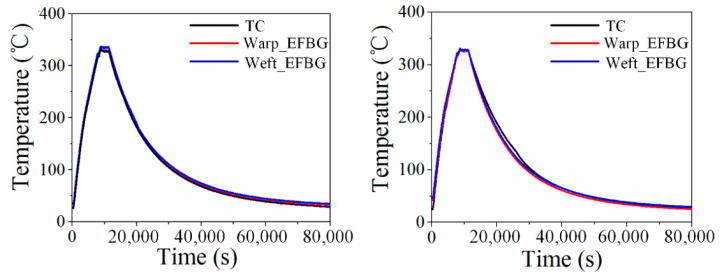
Temperature cycles of the other two repeated experiments.

To eliminate the contingency of the experiment, two additional temperature-monitoring tests were carried out. The results after temperature reconstruction are shown in Figure 11. The average differences between the EFBG and TC measurements in the heating, dwelling, cooling phases, and the whole forming process were 4.41 °C, 2.78 °C, 1.58 °C, and 2.92 °C, respectively. It demonstrates the good performance of the EFBG sensor under harsh conditions of high temperature, a complex woven structure, and high pressure.

## 5. Stress Reflection Using Reflectivity Change

### 5.1. Reflectivity Change 

**Figure 12:**
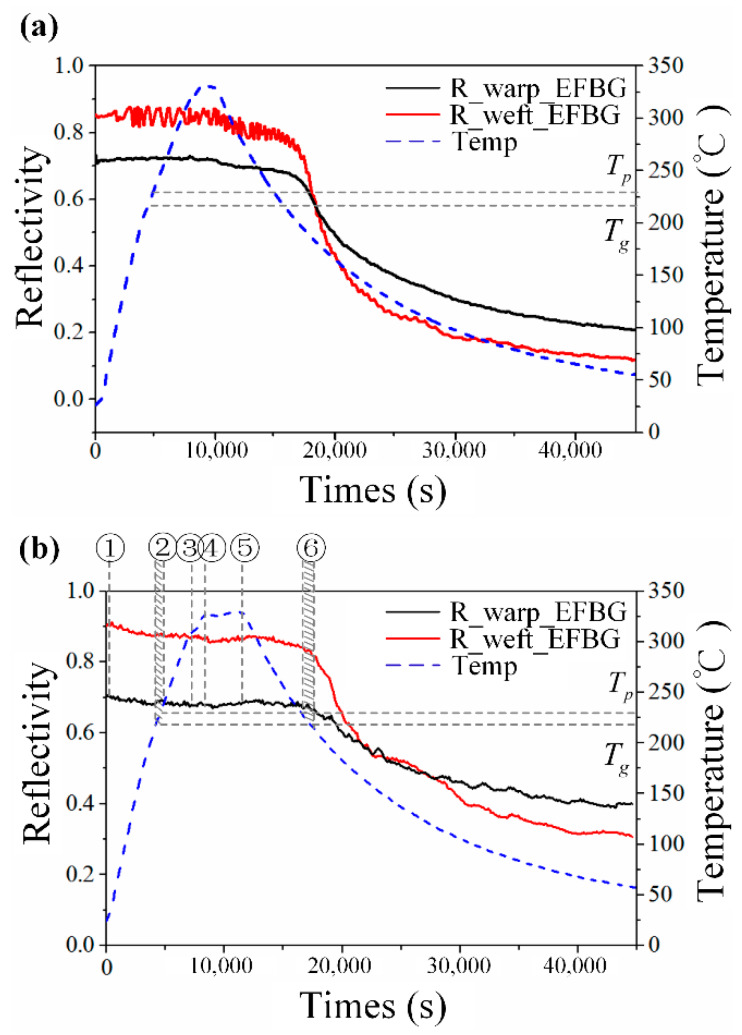
Reflectivity change during the forming process of (**a**) the experiment corresponding to Figure 10 and (**b**) the second set of experiments.

In Figure 10, it is also noticeable that the reflectivity of the spectrum changes, especially in the cooling phase. Figure 12a shows the reflectivity change in the FBGs, along with the warp and weft directions. The micro-bending-caused optical loss was observed before [28,29,30]; however, the reflectivity decrease of an embedded FBG during the cooling phase of the composite forming process has not been comprehensively researched. To clarify the mechanism, the second set of experiment was conducted when the TC was replaced by two strain gauges to record strain change for reference. The reflectivity change in the EFBG sensors in the second experiment, shown in Figure 12b, has a similar trend to that in Figure 12a, demonstrating the repeatability of this phenomenon.

**Figure 13:**
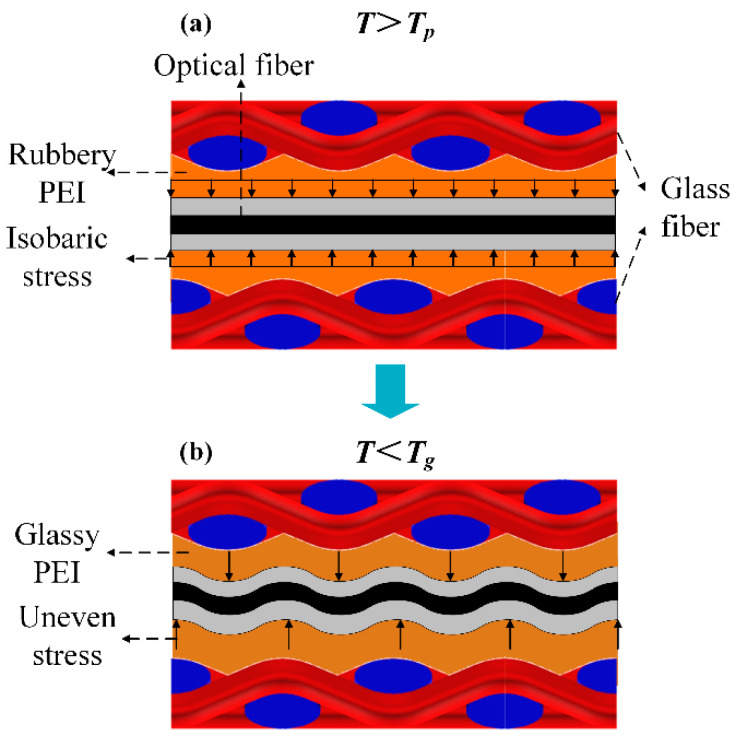
Principle of the bending induced reflectivity change in the pigtail (**a**) above Tp and (**b**) below Tg.

According to the preliminary tests introduced in Section 3, the head of the EFBG can bear 2 MPa without any deformation. Thus, the change in the reflectivity was mainly caused by the bent pigtail. Because there was no pressure applied from Stage 1 to Stage 3, reflectivity during the heating phases barely changed. At Stage 4, although 2 MPa of pressure was applied, the stress around the pigtail was isobaric because the PEI was at its rubbery state and had fluidity, as shown in Figure 13a. Hence, the fiber did not bend, nor did the reflectivity of the sensor change. After the dwelling phase, the temperature started to drop, and PEI entered the leathery state at Stage 6. Although the pressure can be transmitted, the pressure established at different positions relaxed due to the viscoelasticity of the resin [32]. After Stage 6, the temperature is lower than Tg, and the PEI entered the glassy state, at which its Young’s modulus was 2000 times higher than that at the rubbery state [15]. The stress distribution began to vary at different positions, resulting in the appearance of micro-bending, as shown in Figure 13b. This high-frequency longitudinal micro-bending has been validated as the reason for reflectivity decrease [30]. Consequently, the reflectivity change can be reversely used to illustrate the local stress in a composite laminate. This qualitative analysis is also demonstrated by conducting a simple two-dimensional (2D) finite element simulation, as written in the Appendix. In addition, there were decreases in reflectivity of 0.3 and 0.6 for EFBG1 along warp and EFBG2 along weft, respectively, which fits the results in Section 2.2 that the reflectivity reduction along the weft is severer because of the greater warp count than weft count per meter.

### 5.2. Internal Strain Change

**Figure 14:**
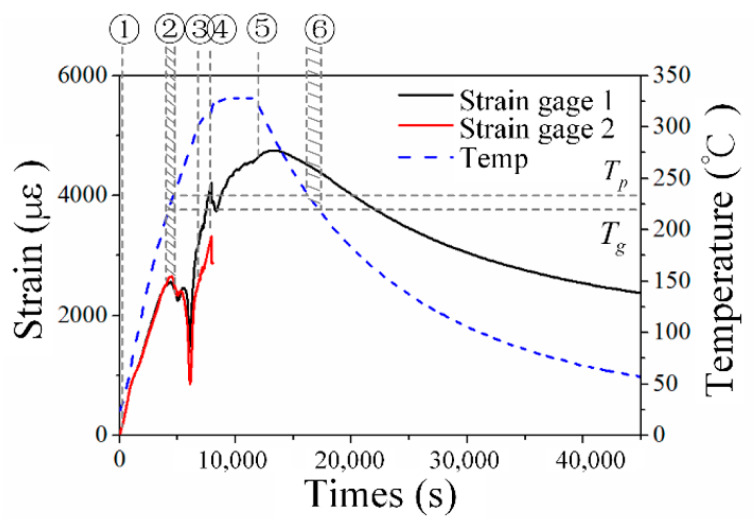
Experimental internal strain.

Figure 14 presents the internal strain change obtained from two strain gauges along the warp direction. One of them became invalid once the pressure was applied because of the uneven pressure induced by the complex microwoven structure. It was found that conventional strain gauge is much more fragile than the newly proposed robust EFBG sensor.

From Stage 1 to Stage 3, as the temperature rose, the internal strain increased due to the thermal expansion of the composite. When the temperature exceeded Tp, Young’s modulus of the PEI decreased rapidly, so the thermal strain could not transmit to strain gauges effectively, and the internal strain suddenly dropped. After 2 MPa of pressure was applied to the composite at Stage 4, the strain increased further because the composite expanded under pressure. Although the temperature started to decrease at Stage 5, the strain continued to reach its maximum value of 4747 με because the expansion caused by compression dominated the thermal shrinkage. After Stage 6, however, characteristic strain changes that had occurred near the glass-transition temperature Tg in the heating phase did not appear.

### 5.3. Comparison of Reflectivity and Strain

Because the cooling phase within the forming process dominates the residual strains and stresses of a thermoplastic composite, the reflectivity of the EFBG smoothed by a Savitzky–Golay filter with a window size of 50 points and the measured strain after Stage 5 were compared and discussed. An analytically calculated strain was also approximated to provide a reference because the strain measured by the strain gauge does not have high reliability. Based on the pressure-volume-temperature results from the preliminary research [33,34], the volume change in the PEI at the temperature profile of the repeated experiment can be estimated using interpolation. Then, the thermally induced strain of the PEI can be calculated as the strain of isotropic material is one-third of its volume shrinkage. By using the temperature-dependent strain of the PEI *ε_m_(T)*, the CTE of the glass fiber *α_f_* in Table 1, temperature profile *T* in Figure 13 and Figure 14, and volume fraction of the fiber *v_f_* that is 50%, the homogenized thermally induced strain of the woven fabric composite can be approximated using the rule of mixture [35].

(3) $$\varepsilon_{L}=\alpha_{f}Tv_{f}+\varepsilon_{m}\left(T\right)\left(1-v_{f}\right),$$

**Figure 15:**
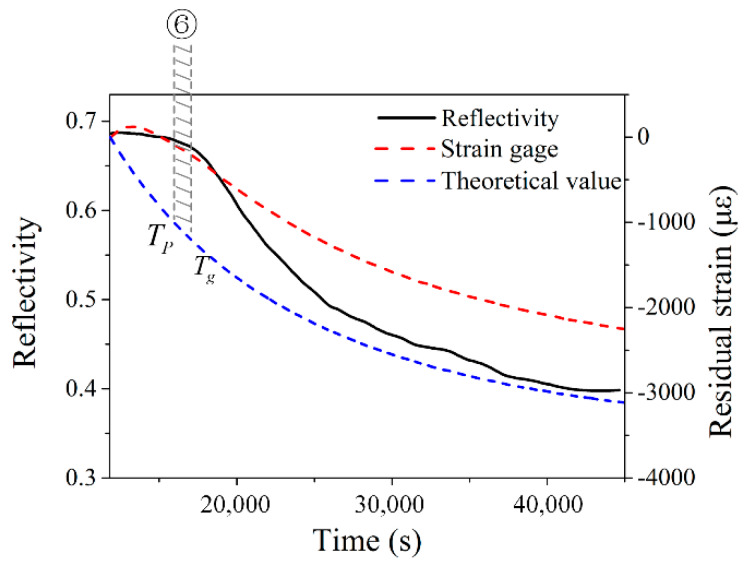
Reflectivity change compared with internal strain.

Figure 15 compares the reflectivity of the EFBG, the measured strain and calculated strain after Stage 5. The strains were all offset to 0 to highlight the change in the cooling phase. Although the woven fabric composite is an anisotropic material with varying thermal expansion along the warp, weft, and out-of-plane directions, the approximate theoretical strain should represent the general trend of strain change [36].

In Figure 15, the curves of the measured and the theoretical strains both decrease in general and have a similar decreasing trend after 13,500 s. This similarity indicates that the in-plane strains, i.e., the strains along with the weft and warp directions, formed once the composite laminate shrank when the temperature began to decrease. The curve of the reflectivity also has a similar trend, although the mechanism of the reflectivity decrease is derived from the micro-bending-caused by the out-of-plane stress. The curve of the reflectivity differs from the other two curves when the temperature starts falling. Before Stage 6, when the temperature is above Tg, the PEI is at its rubbery–leathery stage, which is a ‘stress-free’ stage. The thermally induced stresses only form when the PEI cools down to the glassy stage. The differences between the strain and reflectivity curve indicate that the reflectivity change can illustrate ‘stress’ change rather than ‘strain’. The reflectivity curve indicates when stresses form and how stresses grow directly, which can skip the strain measurement and avoid calculation of multiplying strain and material viscoelasticity that is difficult to determine. Currently, the exact value of stresses still cannot be evaluated, which will be investigated further in future research.

## 6. Conclusions

In this research, the hybrid temperature and stress monitoring of a woven fabric thermoplastic composite could be achieved by using a newly designed FBG based sensing technique. The robust sensor head was manufactured using a steel capillary and high-temperature resistant sealant, and was then pre-annealed at a temperature of 350 °C, leading to the capability of isolating both the axial and transverse strains and withstanding high temperature and high pressure. The nonlinear relationship between the Bragg wavelength shift and the temperature higher than 150 °C was fixed with the cubic polynomial fitting. Then, it is demonstrated that the forming temperature of the woven fabric composite within the heating, dwelling, and cooling phases can be measured by the sensor in both the weft and warp directions, showing only 2.92 °C difference in the temperature measured by the reference TC. It is also found that the microwoven structure of the composite will induce extra loss of the light power along with the optical fiber pigtail when the temperature is lower than the glass-transition temperature of the resin, which can be reversely used to indicate the forming residual stresses according to the reflectivity decrease of the FBG spectrum. In addition, except for a small resin-rich area, the sensor head does not induce severe initial defects in the formed composite due to its small size. The experiments were repeated three times to ensure the reliability of the newly developed technique. 

This experiment explores the changes of temperature and stresses of the woven fabric thermoplastic composite during the forming process, underpins the in situ monitoring technique of embedded sensors in harsh conditions, helps to understand the relationship between the set temperature and actual temperature within a composite laminate during forming, and finds the regularity of residual stresses and reflectivity changes. This new sensor may be also multipliable. For example, EFBG with both lead-in and lead-out lines can be manufactured by bonding both ends of a slightly-bent FBG to the capillary. Multiples of this kind of EFBG sensors can be cascaded to form a sensing network and measure the temperature at multiple points. This EFBG sensor after further development could be used in process monitoring of thermoplastic composite fan blade with high processing temperature and complex residual stresses, and process monitoring of hydrogen composite vessel that has a thick composite layer and complex microstructure.

## Figures and Tables

**Table 1:** Properties of polyetherimide (PEI) and glass fiber.

Parameter	PEI	Glass Fiber
Tensile modulus (MPa)	3276	73,000
Poisson’s ratio	0.36	0.23
Coefficient of thermal expansion (°C^−1^)	5.58 × 10^−6^	5.40 × 10^−6^
Glass-transition temperature Tg (°C)	217	
Rubbery plateau beginning temperature Tp (°C)	227	
Melt index (g/min)	0.42	

**Figure A1:**
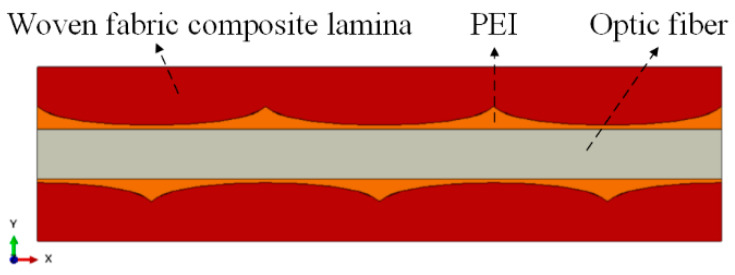
Representative 2D sandwich structure with an embedded optical fiber.

**Figure A2:**
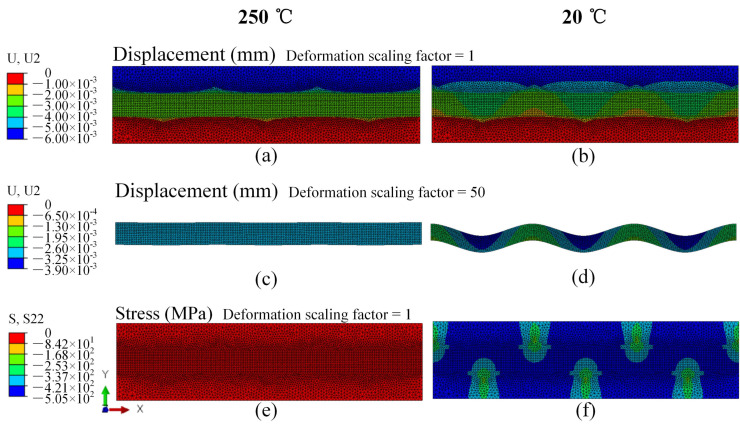
Simulated displacement and stress in the y-direction in the cooling phase. Displacement of the representative model at (**a**) 250 °C and (**b**) 20 °C; displacement of the pigtail at (**c**) 250 °C and (**d**) 20 °C; stress of the representative model at (**e**) 250 °C and (**f**) 20 °C.

## Appendix A

A 2D finite element (FE) simulation of the woven fabric thermoplastic composite cooling from 250 to 20 °C has been conducted using ABAQUS [37]. Figure A1 shows the representative 2D sandwich structure consisting of two composite laminae and an optical fiber in the middle. The gap between the initially straight optical fiber and the woven fabric glass fiber reinforcement is filled with PEI. As preliminary research, PEI and glass fiber are treated as isotropic and elastic [38]. PEI at 250 and 20 °C has Young’s modulus of 7.38 and 4300 MPa, respectively, but has the same coefficient of thermal expansion of 4 × 10^−5^ [15]. The optical fiber is approximated to the glass fiber, given the thin acrylate coating barely influence its mechanical behaviors. The total number of three-node plane stress triangle elements is 11808. Temperature is imposed on the whole model directly as its micro size will have a neglectable temperature gradient. The vertical displacement of the lower boundary is fixed, while the upper boundary is controlled to induce pressure.

The analysis includes two stages. First, a 2 MPa pressure is applied on the top surface when the whole model is at 250 °C (PEI is at its rubbery stage). The left-hand-side column of Figure A2 shows the y-directional displacement and stress. The optical fiber is straight, and the stress is uniform. Then, the y-directional displacement of the upper boundary is fixed after the temperature is lower than Tg (PEI is at its glassy stage) as the upper steel plate of the hot press was also fixed during the cooling phase in the actual experiment. The right-hand-side column of Figure A2 shows the final stage of the model when it is at 20 °C. In Figure A2b,d, the optical fiber that is the pigtail part of the sensor is bent. In Figure A2f, the out-of-plane stress shows a wavy pattern along the fiber direction, and the maximum local stress increases to more than 100 times than that at 250 °C. These FE simulation results show the different optical fiber bending when the PEI is at the rubbery stage and glassy stage, and therefore, qualitatively prove the explanation of the mechanism of optical loss in Section 5.1.
